# Epigenetic regulation of satellite cell fate during skeletal muscle regeneration

**DOI:** 10.1186/s13395-020-00259-w

**Published:** 2021-01-11

**Authors:** Jimmy Massenet, Edward Gardner, Bénédicte Chazaud, F. Jeffrey Dilworth

**Affiliations:** 1 Sprott Center for Stem Cell Research, Regenerative Medicine Program, Ottawa Hospital Research Institute, 501 Smyth Rd, Mailbox 511, Ottawa, ON K1H 8L6 Canada; 2 Institut NeuroMyoGène, Université Claude Bernard Lyon 1, CNRS 5310, INSERM U1217, 8 Rockefeller Ave, 69008 Lyon, France; 3 Department of Cellular and Molecular Medicine, University of Ottawa, Ottawa, ON K1H 8L6 Canada; 4 LIFE Research Institute, University of Ottawa, Ottawa, ON K1H 8L6 Canada

**Keywords:** Muscle stem cells, Regeneration, Epigenetics, Cell fate, Duchenne muscular dystrophy

## Abstract

In response to muscle injury, muscle stem cells integrate environmental cues in the damaged tissue to mediate regeneration. These environmental cues are tightly regulated to ensure expansion of muscle stem cell population to repair the damaged myofibers while allowing repopulation of the stem cell niche. These changes in muscle stem cell fate result from changes in gene expression that occur in response to cell signaling from the muscle environment.

Integration of signals from the muscle environment leads to changes in gene expression through epigenetic mechanisms. Such mechanisms, including post-translational modification of chromatin and nucleosome repositioning, act to make specific gene loci more, or less, accessible to the transcriptional machinery. In youth, the muscle environment is ideally structured to allow for coordinated signaling that mediates efficient regeneration. Both age and disease alter the muscle environment such that the signaling pathways that shape the healthy muscle stem cell epigenome are altered. Altered epigenome reduces the efficiency of cell fate transitions required for muscle repair and contributes to muscle pathology. However, the reversible nature of epigenetic changes holds out potential for restoring cell fate potential to improve muscle repair in myopathies.

In this review, we will describe the current knowledge of the mechanisms allowing muscle stem cell fate transitions during regeneration and how it is altered in muscle disease. In addition, we provide some examples of how epigenetics could be harnessed therapeutically to improve regeneration in various muscle pathologies.

## Background

The development and regeneration of skeletal muscle are mediated by muscle stem cells (MuSCs), also termed satellite cells, that act coordinately to ensure the efficient formation of myofibers while repopulating the niche to allow for repair after future injuries. To mediate myofiber formation, MuSCs must transit through multiple cell fates before achieving their fully differentiated state. Each of these intermediate cell fates share an identical genome that is used as a blueprint for determining their identity. However, the characteristics of each cell fate are determined through alternate interpretation of the genomic blueprint where epigenetic mechanisms are used to determine the subset of genes that will be expressed. These epigenetic mechanisms achieve differential gene expression by controlling the accessibility of the transcriptional machinery to specific loci. Indeed, not all genes within the nucleus can be accessible for gene expression as six billion base pairs of genetic information encoded by human diploid genome must be highly compacted to reside within the confines of the nuclear membrane. DNA compaction occurs through the formation of nucleosomes linking 147 bp and is repeated across the genome at 200 bp intervals with the linker histone H1 protein allowing the protection of inter-nucleosomal DNA. These 10-nm fibers then condense through a disordered self-aggregation to form chromatids [1]. While the DNA organization within each cell has been suggested to be unique [2], general rules emerge. For instance, genes that are not expressed tend to be associated with the nuclear periphery while transcribed genes tend to aggregate in the nuclear lumen. This distinct nuclear organization between cells is the basis for establishing and maintaining cell-specific gene expression programs. The chromatin state is fluid to allow expression of the needed genes in a spacio-temporal manner. One mechanism allowing fluidity of the chromatin structure is the dynamic nature of nucleosome association with DNA where nucleosome displacement allows incorporation of unmarked histones within newly formed nucleosomes. This histone exchange represents more than a simple turnover mechanism, as canonical histones in nucleosomes can be replaced by different histone variants. Histone variants alter the chemical properties of nucleosomes and change their stability.

Ability to alter chromatin organization is critical for mediating decisions of cell fate and differentiation. In response to environmental cues, chromatin organization is modified and alters the accessibility of the transcription machinery to the gene sequences, which is modulated by several levels of regulation. The first level lies within the DNA itself where the presence of specific sequence elements surrounding the gene can act as promoters or enhancers that serve as binding sites for transcription factors (TFs), which can recruit the transcriptional machinery to locus. The ability to recruit the transcriptional machinery may be increased by promoter-enhancer interactions that can occur through chromatin looping to modulate gene expression. A second level of regulation is established through differential accessibility of TFs or the transcription machinery to DNA elements. This can be performed by modulation of chromatin conformation, caused by reversible modifications of either DNA or histones through epigenetic processes.

DNA methylation is a widely used epigenetic mechanism to regulate chromatin accessibility. The predominant DNA modification in mammals is CpG methylation, where the addition of a methyl group at carbon 5 of the deoxyribonucleotide cytosine alters the affinity of the TF for its DNA element. CpG methylation stabilizes gene silencing through two potential modes: (I) blocking access of DNA binding proteins required for transcription by impairing their ability to bind DNA elements or (II) attraction of TFs containing methyl-CpG-binding domains that are able to repress transcription. It has been proposed that DNA methylation does not trigger gene repression but instead stabilizes repression at already silenced genes [3].

In contrast to the limited number of characterized DNA modifications, histones undergo a wide-variety of post-translational modifications (PTMs) including acetylation, methylation, phosphorylation, ubiquitination, ADP-ribosylation, and citrullination. Among them, some histone PTMs are known to allow chromatin compaction while others direct chromatin decompaction. For instance, trimethylation of lysine 9 and lysine 27 of histone 3 (H3K9me3 and H3K27me3) or trimethylation of lysine 20 of histone 4 (H4K20me3) are associated with local chromatin compaction. This compaction modulates TF or RNA polymerase II access to target sequences and leads to the repression of gene expression. In contrast, acetylation of lysine 9 of histone 3 (H3K9Ac) and lysine 20 of histone 4 (H4K20Ac) and the trimethylation of the lysine 4 of histone 3 (H3K4me3) lead to relaxation of the chromatin state, improving accessibility of the transcription machinery and increasing local gene expression [4–6]. Presence of relaxed chromatin at gene enhancer caused by monomethylation of lysine 20 of histone 3 (H3K20me1) and acetylation of lysine 27 of histone 3 (H3K27Ac) also modulates gene expression [7, 8]. Thus, the relationship between histone modifications and transcriptional output reveal how the epigenetic code regulates gene expression.

In the present review, we will discuss the role for epigenetic regulators in mediating the regenerative function of MuSCs in adults. The role for diseased and aged muscle environment in modifying the epigenetic landscape of MuSCs will also be examined.

## MuSCs mediate regeneration of skeletal muscle

Residing at the periphery of the muscle fiber between the sarcolemma and the basal lamina [9], MuSCs lay dormant in a quiescent state, ready to respond to muscle injury. The fate of MuSCs is regulated by a series of TFs that include Pax7 and a family of myogenic basic-Helix-loop-helix proteins, termed the myogenic regulatory factors (MRFs), that include MYOD, MYF5, MRF4, and myogenin (MYOG) proteins [10–12]. These muscle TFs work with other, more ubiquitous TFs, to establish the epigenetic states to regulate muscle regeneration.

**Fig. 1:**
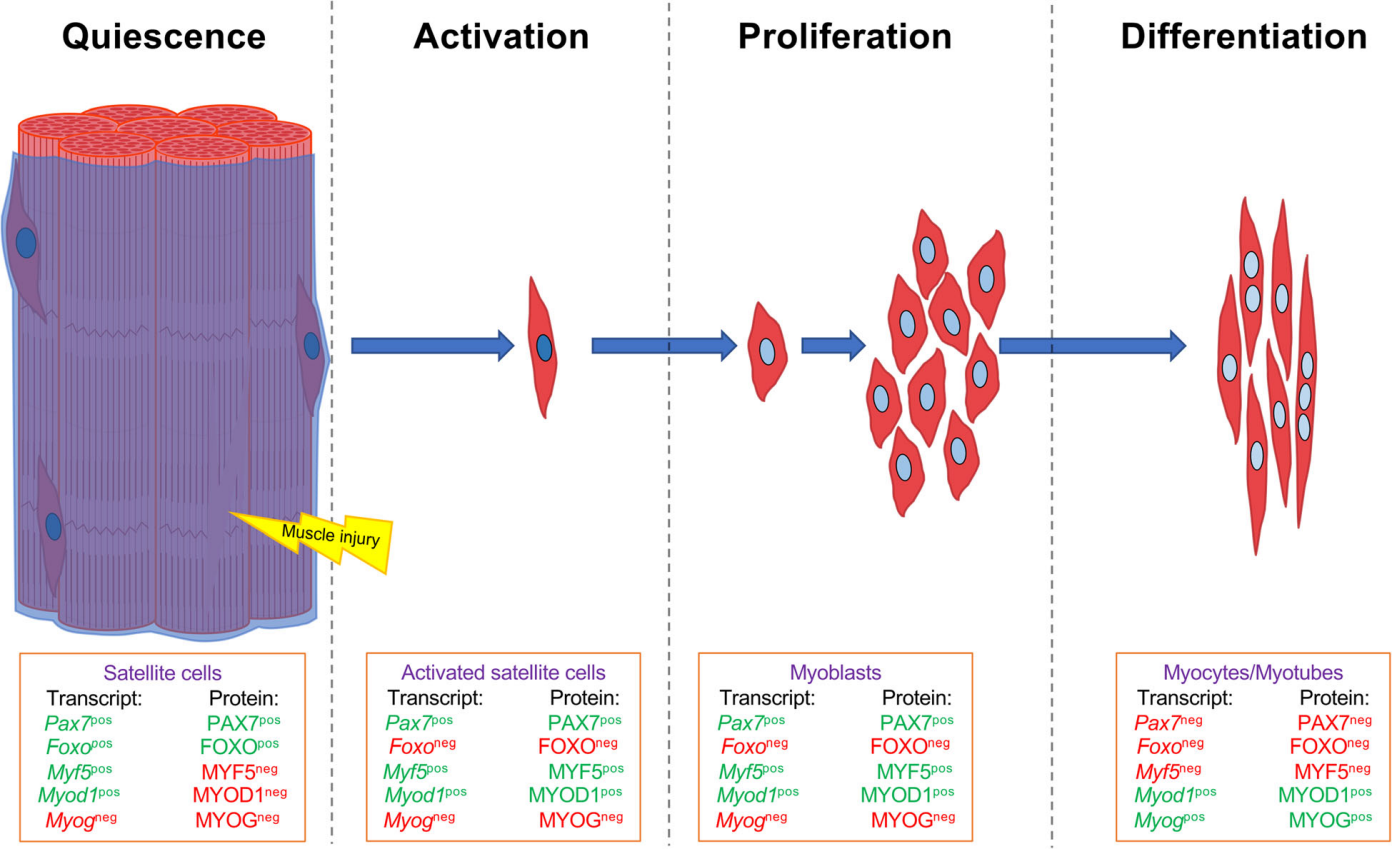
Hierarchy of TF expression during muscle regeneration process. After a muscle injury, muscle stem cells (MuSCs) are activated and exit the quiescence state. Activated MuSC transit to proliferative muscle progenitor cells (myoblasts) which next transit into differentiated myocytes. Myocytes are able to fuse to each other into myotubes, or to newly formed myofibers in order to restore the damaged muscle. In green, expressed genes or proteins; in red, unexpressed genes or proteins

MuSC quiescent state is characterized by the expression of PAX7 and FOXO transcription factors [13]. *Myf5* and *Myod1* are also transcribed in quiescent MuSCs, but post-transcriptional regulation prevents their translation (PAX7^+^FOXO^+^MYF5^-^MYOD1^-^) (Fig. 1) [13, 14]. More specifically, *Myf5* mRNA is sequestered in messenger ribonucleoprotein granules (mRNPs) to avoid its translation [15] while *Myod1* mRNA retains an intron that prevents its translocation out of the nucleus [16]. After muscle injury, MuSC activation leads to symmetric and/or asymmetric division. Asymmetric divisions produce a PAX7^+^MYF5^+^ cell, destined to the myogenic program and a PAX7^+^MYF5^-^ cell that repopulates the pool of quiescent MuSCs. Symmetric division produces two identical daughter cells: division of Pax7^+^Myf5^-^ cells expands MuSC pool and division of more committed PAX7^+^MYF5^+^ expands that of MuSCs [17]. Activated MuSCs exhibit a PAX7^+^FOXO^-^MYF5^+^MYOD^+^ protein expression profile. To achieve this state, *Myf5* and *Myod1* mRNAs begin to be translated, while PAX7 expression decreases and FOXO ceases to be expressed. Activated MuSCs are now poised to rapidly expand their population through continued cell cycle progression in response to environmental signaling cues in the damaged muscle. As the MuSCs start to accumulate, PAX7 and MYF5 expression becomes repressed while MYOG becomes expressed to drive cell cycle exit and form myocytes (PAX7^-^FOXO^-^MYF5^-^MYOD1^+^MYOG^+^) (Fig. 1) [13, 18]. Finally, the formation of multinucleated myofibers results in the decreased expression of MYOD1 while MYF6/MRF4 becomes highly expressed in the functional muscle fiber [19–21].

The role for MRFs in controlling MuSC fate is well established. However, the myogenic TFs require coordination with a broad range of transcriptional regulators that help modulate the epigenetic landscape that controls specific gene expression programs. Below, we will discuss the different epigenetic factors that work in coordination with the myogenic TFs to facilitate transitions in cell fate. We will discuss both in vitro and in vivo observations, keeping in mind that studies carried out in vivo are inherently more difficult to interpret as the signaling cues can be derived from other cell types within the regenerative muscle environment. Unless otherwise stated, studies discussed below were performed in vitro.

## Epigenetic regulation of adult myogenesis

### Quiescence and early activation

Quiescence is a state where cells enter into a reversible cycle arrest in G_0_ phase of the cell cycle. Studies investigating the role of chromatin and epigenetic regulations in the maintenance of MuSC quiescence have largely been performed on MuSCs isolated from uninjured muscle, with the assumption that cells retain the characteristics of a quiescent cell during the sorting procedure. However, recent work has shown the importance of isolation protocols in the study of true quiescence MuSCs [22]. Indeed, the development of in situ fixation techniques to lock cells in a quiescent state prior to isolation has exposed important differences in gene expression as well as histone post-transcriptional modifications. Extensive changes in epigenetic marks on histone H3 are observed during the 3-h period needed to isolate MuSCs, though no differences in DNA methylation were observed within this time frame [16, 22, 23]. Based on these findings, one can assume that most of the epigenetic information collected using isolated MuSC analysis do not represent the quiescent state but a transition between quiescence and activation known as early activation [24, 25].

Maintenance of the quiescent state requires repression of the genes coding for both cell cycle proteins and permanent cell cycle exit. p53 was shown to maintain a reversible cell cycle arrest in quiescent MuSCs whereas the activation of tumor suppressor ARF (p16^INK4a^) leads to a definitive cell cycle arrest and senescence [24, 26]. To maintain this balance, different pathways contribute to the quiescent MuSC transcriptional network. In particular, the expression of forkhead box (FOXO) transcription factors, FOXO1, FOXO3A, and FOXO4, were reported to be required for the maintenance of the MuSC quiescence [13]. FOXO3 maintains the expression of Notch pathway components [27]. Active Notch pathway leads to a decreased expression of MDM2 which allows accumulation of p53 to maintain cell cycle arrest and quiescence until injury [26–28]. On the other hand, *p16*^*INK4A*^ needs to be kept in a repressed state to prevent MuSCs from entering into a definitive senescent cell cycle arrest [26].

In the quiescent state, transcription levels are relatively low due to the condensed state of the chromatin [29]. Nevertheless, many genes are expressed including MRFs which show accumulation of mRNA but not of their encoded proteins. This shows that MuSCs use additional mechanisms beyond transcriptional regulation to modulate their fate as it is the case for *Myf5* and *Myod1* mRNA nuclear retention. That being said, *Myod1* expression levels must be minimized. This reduction must be controlled through epigenetic mechanisms. As a matter of fact, expression of a H4K20me2 methyltransferase, Suv4-20 h1, is required for condensation of chromatin and repression of *Myod1* expression in quiescent MuSCs [29, 30].

### Histone post-translational regulation of MuSC quiescence and activation

Though the expression of PAX7 and FOXO transcription factors are key features of quiescent MuSCs, it is unclear how their expression is controlled during quiescence.

In activated MuSCs, *Pax7* expression is regulated by the antagonism of Polycomb (PcG) and Trithorax (TrxG) group proteins to silence or activate its expression, respectively. Indeed, the lysine methyltransferase MLL1 (a Trithorax group sub-unit) KO mice see a loss of *Pax7* expression in activated and proliferating MuSCs (by the addition of H3K4me3 marks at its promoter) while having no effect on *Pax7* expression in quiescent MuSCs [31].

To maintain quiescence, MuSCs prevent cell cycle entry through the expression of specific cell cycle inhibitors. As mentioned above, the choice of cell cycle inhibitors is indispensable, as the expression of p16^INK4a^ cell cycle inhibitor leads to senescence and permanent cell cycle exit. The repression of p16^INK4a^ in MuSCs is assured by the Polycomb PRC1 complex where the Ring1B E3 ubiquitin ligase mediates H2A monoubiquitination at lysine 119 (H2AUb) of the *INK4a* locus [26, 32, 33]. In addition, the Polycomb PRC2 complex containing the EZH2 subunit was shown to bind at the *INK4a* promoter to control its transcription in in vitro culture of mouse embryonic fibroblasts through the depositing of the repressive H3K27me3 mark [34]. Regulation of the *INK4a* locus by PRC2 is also likely to occur in MuSCs as a MuSC-specific KO of EZH2 prevented the expansion of the stem cell population [35].

MYOD1 has an important function in MuSC commitment, and its expression is needed for MuSC activation and proliferation. While the gene is expressed at low levels, a repressive chromatin environment is maintained at the *Myod1* locus to prevent high-level expression until activation. Histone methyl transferase (HMT) Suv4-20 h1 adds H4K20me2 marks at *Myod1* promoter and at its distal regulatory region (DRR), 5 kb upstream of *MyoD1* transcription starting site. Addition of H4K20me2 at these sites induces heterochromatin formation and decreased *Myod1* expression in early activated MuSCs [29]. Since this mechanism is necessary to repress *Myod1* expression during quiescence too [29, 30], one can assume that these histone PTMs are already present at the quiescent state and maintained during the early activation. The DRR and promoter of *Myod1* are also marked by the repressive H3K9me2 modification. While the enzyme responsible for H3K9me2 marking is not known, some mechanisms maintaining this mark have been uncovered. The E3 ubiquitin ligase Deltex2 is essential to maintain H3K9me2 mark through the inhibition of lysine demethylase jumonji domain containing 1C (JMJD1c) enzyme [36]. Inhibition of JMJD1c functions prevents demethylation to allow maintenance of H3K9me2 marks at the *Myod1* promoter and DRR. It is noted that the removal of H3K9me2 at these regulatory regions is necessary for the increased expression of *Myod1* that drives MuSC activation [36].

Finally, the expression of muscle-specific genes is also repressed in quiescent and activated MuSCs. In this case, the PRC2 complex mediates the addition of H3K27me3 marks to myosin heavy chain 2b (*Myh4)* and *Myogenin* (*Myog)* promoters and to muscle creatine kinase gene (*MCK)* enhancer, leading to their repression of expression [37]. While this level of regulation is inferred from early activated MuSCs, it will be important to confirm whether PRC2 is also present at these muscle-specific genes in true quiescent MuSCs.

### The DNA methylation landscape during MuSC quiescence and early activation

Technical limitations have hindered our understanding of functions of DNA methylation in the maintenance of MuSC quiescent state. One could infer that the DNA is methylated at the MyoD locus to prevent transcription based on the original lineage conversion studies in fibroblast cell lines [38]. However, studies in primary fibroblasts have shown that the MyoD locus is not methylated in normal conditions and only becomes methylated as part of a genome-wide increase in CpG island methylation in response to crisis [39]. Thus, the role for DNA methylation at MyoD and other genes in regulating the transition between satellite cell quiescence and activation is an area that still needs to be explored and will be facilitated as new technologies that allow analysis of DNA methylation on a small number of cells and improvement of techniques to isolate and study quiescent MuSCs become available.

### Epigenetic histone modifications contributing to the proliferative state of MuSCs

MYF5 is a key transcription factor contributing to the proliferation of MuSCs. The *Myf5* gene is already expressed in quiescent MuSCs but, upon activation, trimethylation of H3K4 (H3K4me3) at its promoter leads to an increase of its expression. Marking of the *Myf5* promoter by H3K4me3 is mediated by the HMT complex, WDR5/ASH2L/MLL1 [40]. This HMT complex is recruited by PAX7 through an interaction that requires methylation of PAX7 by the CARM1 protein [41, 42]. HMT recruitment at *Myf5* underlies the importance of PAX7 expression for activation and proliferation of MuSCs. In the same context, MLL1 KO also displays diminution of *Myf5* gene and protein expression in proliferating primary myoblasts and C2C12 cells [31, 43]. These effects observed in proliferating myoblasts are consistent with the fact that *Pax7* expression needs to be maintained during the transition from quiescence to proliferation and during the proliferation. Maintaining the open state of chromatin at the *Pax7* gene is attributed to a direct effect of the switch/sucrose nonfermentable chromatin remodeling complex (SWI/SNF) chromatin remodeling complex. Indeed, the Brg1 SWI/SNF subunit is phosphorylated by casein kinase 2 and contributes to the formation of SWI/SNF complex at *Pax7* gene, promoting its expression and leading to MuSC proliferation [44, 45].

**Fig. 2:**
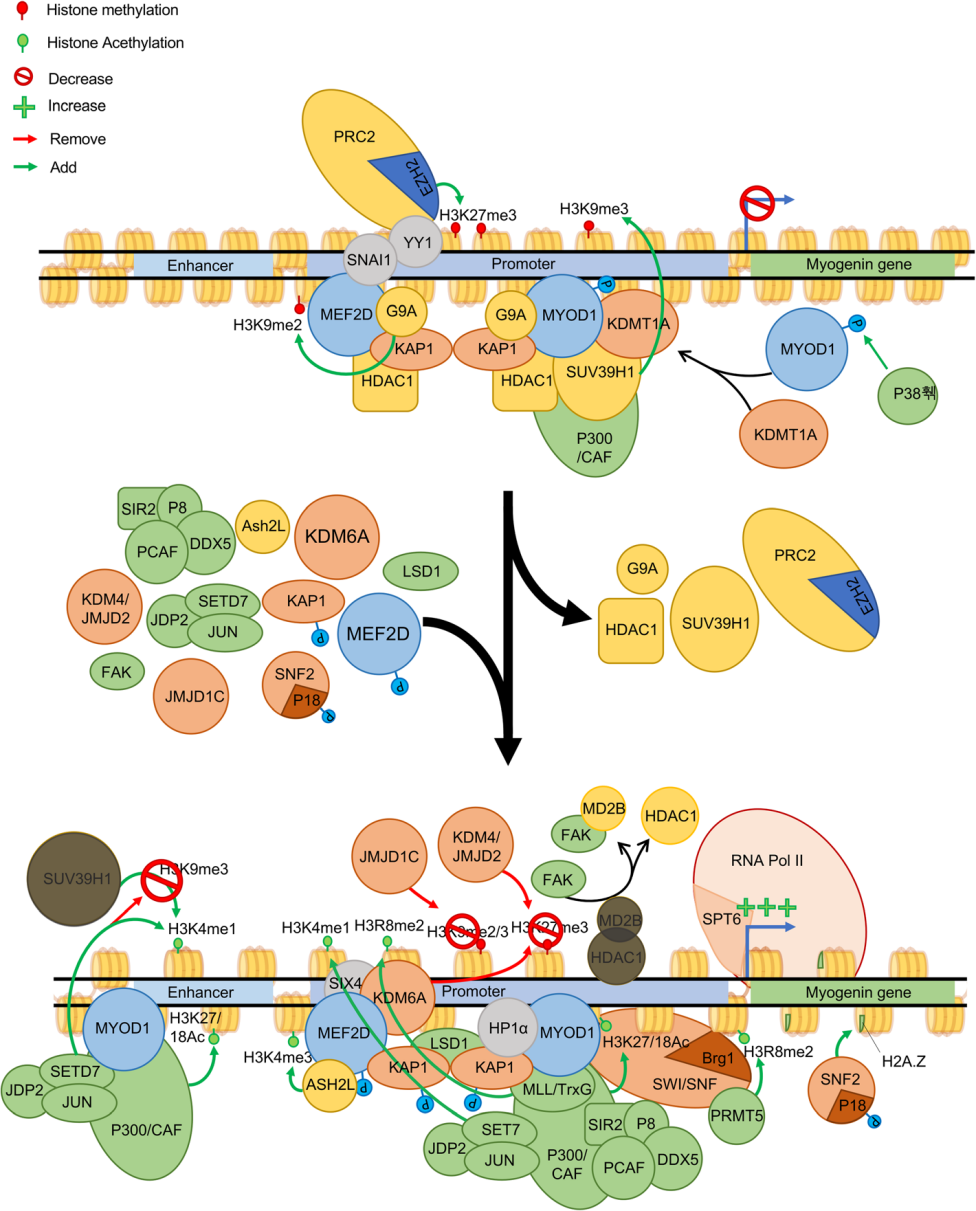
Regulation of the *myogenin* gene control the transition from proliferative myoblasts to differentiated myocytes. To block MyoG expression and prevent early differentiation, a repressive function of MYOD1 is needed. In this repressive action, MYOD1 is recruited on the promoter and is bound by KDMT1A thanks to P38γ phosphorylation at its Ser199 and 200. MYOD1 forms a poised complex with MEF2D, KAP1, G9a, and HDAC. Histone acetyltransferase P300/CAF proteins can also bind to MYOD1/MEF2D complex, but their functions are limited. During induction of differentiation, MYOD1 functions change to allow *Myog* gene expression. This transition is due to phosphorylation of KAP1, which leads to removal of HDAC1 and G9a proteins from MYOD1/MEF2D complex. In this state, P38α phosphorylates MEF2D at its threonine 308 and 305, which leads to the recruitment of ASH2L and trimethylation of H3K4. At the same time JDP2, JUN and SETD7 are recruited at p300/CAF proteins to allow the establishment of permissive marks H3K4me1 and H3K27/18Ac at the enhancer of *Myog* and the H3K4me1 mark works as an antagonist to the addition of H3K9me3 by SuV39h1. At the promoter, the MLL/TrxG complex is recruited by MyoD to dimethylate H3R8. In addition, the repressive marks H3K9me2/3 and H3K27me3 are removed by JMJD1c and JMJD2 (KDM6A), respectively, while permissive marks, H3K4me3, H3K36me3, H3R8me2, and H3K27/18Ac, are added at the promoter by MLL2, SETD2, PRMT5, and p300. The presence of these permissive marks allows the recruitment of RNA polymerase II and starting of transcription. At the *Myog* gene, P38α is responsible of the phosphorylation of MEFD2 as well as of P18 Hamlet (a subunit of SNF2); this phosphorylation leads to the incorporation of unstable H2A.Z variant at the core gene to facilitate transcription

In proliferating MuSCs, PAX7 is recruited to areas of open chromatin and its presence correlates with active histone marks H3K4me1 and H3K27Ac. In particular, PAX7 facilitates chromatin accessibility at gene loci encoding MRFs through activation of transcriptional enhancers [46]. Among these MRFs, MYOD1 plays a key role in regulating both proliferation and differentiation. In proliferating cells, the Msh homeobox 1 (MSX1) TF was shown to bind a mouse specific isoform of histone H1, H1b, at the core enhancer region (CER) of *Myod1* in order to induce chromatin compaction and to reduce *Myod1* expression [47]. Additionally, histone deacetylases (HDACs) are known to be necessary for the maintenance of proliferation, and class IIA HDACs, HDAC4 and HDAC5, are recruited by the H3K9me3 methyltransferase SUV39H1 to specifically target *Myod1* promoter. In this way, they modulate *Myod1* expression, which underlines the importance of chromatin shape at *Myod1* gene for maintenance of proliferation or entrance in differentiation (Fig. 2) [48]. The accumulation of the repressive epigenetic factors at the *Myod1* gene in proliferating MuSCs is modulated in response to Notch1 signaling to prevent differentiation where expression of *Myod1* oscillates due to transient expression of the Notch-regulated transcriptional inhibitor HES1 [49, 50]. Lahmann et al. showed that decreased HES1 expression leads to maintenance of MYOD expression and differentiation [50]. These mechanisms likely work in tandem to orchestrate temporal control of the transition from proliferation to differentiation.

Though expressed at lower levels in proliferating cells, MYOD1 contributes to both proliferation and differentiation. MYOD1 ensures the repression of muscle differentiation genes during proliferation and then the activation of the same genes in response to differentiation cues [12]. A role for MYOD1 in both proliferation and differentiation may seem contradictory but is readily understood when one considers that this TF can be both a repressor and an activator at specific genes depending on the context. In this model, MYOD1 and MEF2D interact with the scaffold protein KAP1 [51]. In proliferation conditions, MYOD1, MEF2D, and KAP1 act together to stabilize the association of both co-repressors (G9A, HDAC1) [48, 52] and coactivators (P300 and LSD1) [53, 54] at the muscle differentiation genes. The assembly of this enhanceosome-type complex establishes a poised chromatin state at the promoters where the repressive enzymes dominate to limit gene expression. During differentiation, the increased expression of mitogen-activated protein kinase (MAPK) P38α (P38α) leads to activation of MSK1 kinase which phosphorylates KAP1 at serine 473 [51]. The phosphorylated KAP1 protein no longer interacts with the co-repressors, but continues to maintain an interaction with the coactivators, leading to the establishment of an open chromatin state and the expression of muscle target genes (Fig. 2) [51]. In other words, when both co-repressors and coactivators associate with MYOD1, the co-repressors dominate. Signals from the environment induce phosphorylation by MSK1 that in turn displaces the co-repressors from the locus [51]. MYOD1 contribution to MuSC proliferation directly suggests the possibility of regulation of *Myod1* expression level during proliferation.

Additional co-repressors are associated with the MYOD1-MEF2D-KAP1 complex in proliferating cells. Heterochromatin proteins HP1α and β interact with MYOD1-MEF2D-KAP1 to repress its activity at target gene promoters to maintain proliferation [55]. Similarly, the formation of a HDAC1/MYOD1-KAP1 complex allows deacetylation of MYOD1 target genes. One of their targets, *Myog*, which is necessary for differentiation, shows reduced acetylation and a recruitment of SUV39h1 thanks to the presence of HDAC1/MYOD1 complex. In C2C12 cell line, the formation of another complex containing MYOD1/P300/CBP-associated factor (PCAF) and the HDAC SIRT2 is necessary to maintenance of proliferation and repression of differentiation [56]. Such as other SIRT enzymes, SIRT2 is dependent of the level of NAD+, revealing the implication of the metabolism for the maintenance of proliferation. The removal of histone acetylation and the recruitment of Suv39h1 leads to an enrichment of H3K9me3 marks and maintenance of the chromatin under a closed state, leading to repression of *Myog* expression and impeding the start of differentiation (Fig. 2) [57, 58]. Another study revealed the function of P38γ MAPK into the phosphorylation of the Ser199 and 200 of MYOD1 to allow the recruitment SUV39h1/KMT1a to the *Myog* promoter, reducing its expression during proliferation [59]. Moreover, SNAIl family transcriptional repressor 1, SNAI1, associated with HDAC1/2 can bind E-box at differentiation-related genes and prevents the binding of MYOD1 and target gene expression [60]. This mechanism suggests the importance of the SNAI1/HDAC1/2 complex in promoting proliferation by blocking MYOD1 from initiating differentiation.

**Fig. 3:**
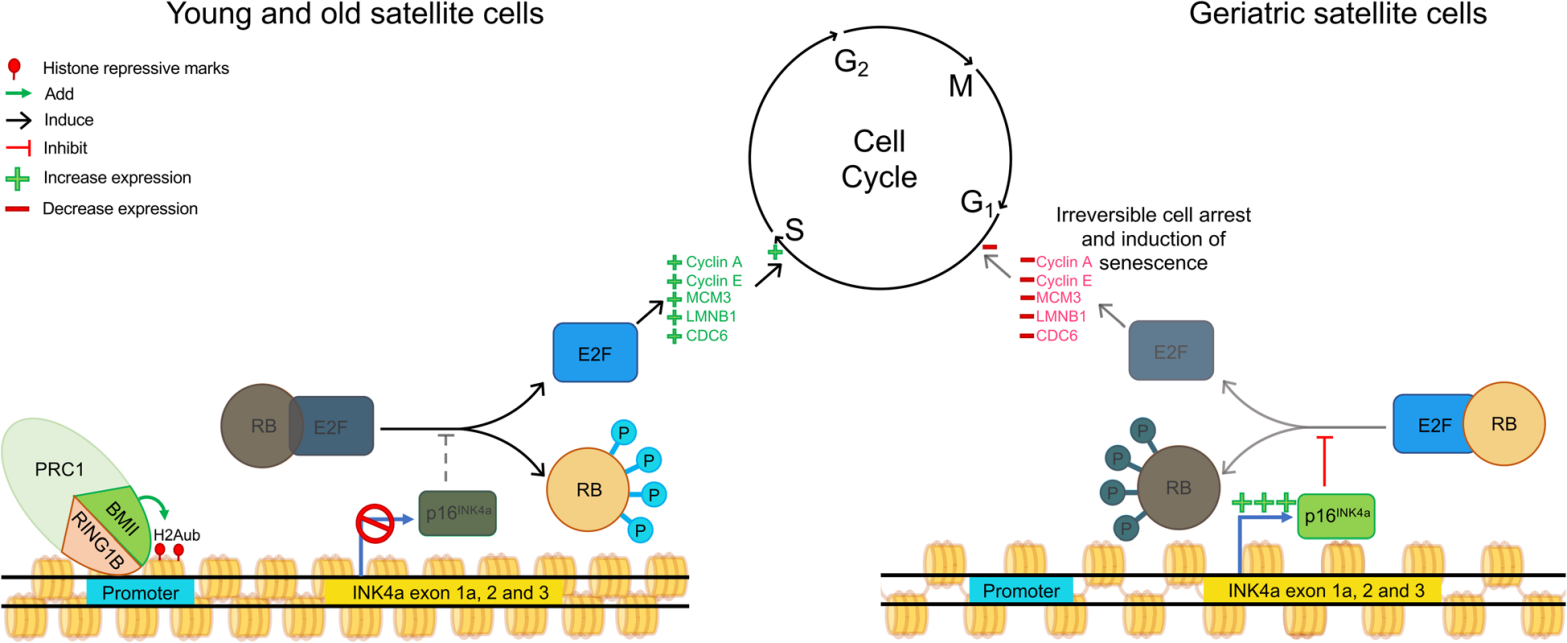
Senescence mediated by irreversible cell cycle arrest caused by p16^INK4a^ expression in sarcopenic MuSCs. In young and old MuSCs, the presence of PRC1 complex containing RING1 and BMI1 subunits leads to H2A ubiquitination at the promoter of INK4a locus and the repression of p16^INK4a^. This repression allows RB protein phosphorylation and loss of stability of E2F/RB complex. Once free from its complex, E2F induces the expression of genes promoting the cell cycle. Sarcopenic MuSCs lose their PRC1 repression of p16^INK4a^, leading to reduction of RB phosphorylation and maintenance of the E2F/RB complex. Without free E2F protein, the expression of genes promoting cell cycle is reduced, causing cell cycle arrest and senescence of MuSCs

To maintain myoblast proliferation, repression of differentiation is not enough. Cells must also maintain the expression of genes involved in cell cycle progression. Like in most cell types, E2F protein family plays a key role in regulating cell cycle through the recruitment of histone acetyltransferase P300/CBP and PCAF/GCN5 histone acetylases to the cyclin genes [61, 62]. In primary mouse myoblasts, the E2F1/PCAF complex mediates acetylation of histones at E2F1 target genes to allow passage through the G1/S cell cycle checkpoint [63]. In addition to the recruitment of acetyltransferase, studies in many different cell types have shown that E2F proteins mediate recruitment of H3K4 histone methyltransferases from the KMT2 family [64]. In the muscle system, proliferating C2C12 cells utilize MLL5 to deposit H3K4me3 marks at the cyclin A2 gene, a factor necessary for progression through G1/S cell cycle checkpoint [65]. Finally, H3K36me methyltransferase SET2 KO in C2C12 cells compromise G1/S and G2/M phase transition by decreasing levels of cyclin D1, CDK4, CDK6, and cyclin E2. This result indicates an essential function of the H3K36 methyltransferase SET2 for the maintenance of myoblast proliferation [66]. Thus, it is clear that epigenetic modifications of histones contributes to maintain the cell cycle progression though we still lack an in depth understanding of all the players involved in myoblast expansion.

The degree to which genes are marked by histone post-translational modifications depends not only on the presence of epigenetic enzymes, but also on the availability of key co-factors that contribute to their enzymatic activity. Indeed, the energy source available to the cell will affect the availability of these co-factors. The glycolytic environment of proliferating MuSCs ensures an abundance of several intermediates of the tricarboxylic acid cycle (TCA) that are needed for establishment of epigenetic modifications. In particular, oxidative decarboxylation of pyruvate supplies the acetyl-CoA donor of acetyl utilized by the HAT proteins [67]. Similarly, the metabolite α-ketoglutarate is a necessary co-factor for the demethylation of either DNA or histones, by ten-eleven translocation (TET) and JMJD proteins, respectively. The functions of the histone demethylase LSD1 and the HDACs also depends on the availability of FAD+ and NAD+ in the cell [68]. Independently of the TCA, the intracellular methionine is transformed in S-adenosylmethionine which is necessary for the action of DNA methyltransferase (DNMT) proteins [68]. Understanding these points highlight the importance of the metabolism in the epigenetic regulation of the MuSC. Indeed, after isolation of mouse MuSCs, during the transition from quiescence to proliferation, a shift between fatty acid oxidation and glycolysis occurs [69]. This shift induces a decrease of NAD+ levels which reduces the activity of SIRT1 family of NAD+-dependent HDAC enzymes. The activity of SIRT1 is associated with a regulation of H4K16Ac marks associated to muscle gene expression [69]. While characterization of metabolic pathways contributing to the epigenetic regulation of MuSC proliferation remains in its infancy, new technologies in the area of metabolomics make this an exciting area of current research.

### The DNA methylation of proliferating MuSCs

DNA methylation plays a key role in preventing the expression of cell cycle inhibitors during MuSC proliferation. Among them, repression of cyclin-dependent kinase inhibitor 1C (CDN1C or P57kip2) is required to prevent differentiation. Interestingly, deleting DNA methyltransferase DNMT3A in MuSCs leads to a diminution of proliferation that correlates with increased expression of the *Cdkn1c* gene and decreased DNA methylation at its promoter sequence. This loss of proliferation in the DNMT3A KO myoblasts can be rescued with *Cdkn1c* KD, indicating an important function of de novo methylation in the maintenance of MuSC proliferation [70]. The control of *Cdkn1c* DNA methylation can occur indirectly through MYOD1 functions. Indeed, MYOD1 has been shown to control the expression a transcriptional repressor Zinc Finger Protein 238 (ZFP238) in C2C12 cells [71]. This is significant as the ZFP238 protein is able to recruit DNMT3A and HDAC1 at the promoter of myogenic genes to repress their expression [72]. It is possible that in MuSCs, ZFP238 is also present at *Cdkn1c* promoter and recruits DNMT3A. At the moment, there is no work to support these hypothesis and analysis of the presence of ZFP238 and DNMT3A at the *Cdkn1c* promoter has to be investigated.

The continued proliferation of myoblasts is also dependent on repression of *Myog* gene expression to prevent differentiation. This repression was shown to be possible thanks to the methylation at the *Myog* promoter [73, 74]. The *Myog* promoter has only 1.4% of CpG dinucleotides. However, even if the region is not rich in CpG island, bisulphite and methylation-sensitive restriction endonuclease analysis revealed a hypermethylation state during proliferation of C2C12 cells, necessary for *Myog* repression [73]. Additionally, methylation of the scattered CpG sites is necessary for the binding of a methyl-CpG-binding protein, ZBT38 (CIBZ). The presence of ZBT38 at CpG islands within the *Myog* promoter acts to inhibit its expression and maintenance of proliferation as the knockout of ZBT38 leads to differentiation of C2C12 cells [75].

Taken together, these findings show that as MuSCs start to proliferate, TFs target the epigenetic machinery to genes necessary to ensure cell cycle progression and those preventing differentiation.

### Epigenetic regulation of the differentiation process

After the expansion phase, MuSCs undergo cell cycle arrest and transit towards differentiation [76]. Cell cycle arrest is initiated through the expression of the cell cycle inhibitors CDKN1C or CDKN1A (p21) [77]. Moreover, a progressive increase of MYOD1 protein expression leads to the increased expression of early differentiation markers such as *Myog* and coincides with the abrogation of MYF5 and PAX7 expression [78]. Coincident with the onset of differentiation, MuSCs are marked by hyperacetylation of histones H3 and H4 while decreased methylation of H3K9 and K27 is observed [79, 80]. The epigenetic reprogramming of MuSCs allows the opening of chromatin at specific muscle loci needed for differentiation.

### The control of MuSC differentiation by histone post-transcriptional

The differentiation of human myoblasts has been shown to result in a large change in the histone PTM landscape with a global diminution of H3K9me3 and H4K20me3 repressive marks. In particular, H3K9me3 is erased from *Myod1* and *Myog* loci [79]. These results point to the importance of histone modification modulation for myoblast differentiation.

One of the first steps during the transition to differentiation is the stop of MuSC proliferation by cell cycle arrest. In this context, the downregulation of *Pax7* expression must occur. Indeed, PRC2 interacts with Yin Yang 1 TF (YY1) via the phosphorylation of the threonine 372 of EZH2, a subunit of PRC2 complex, by p38α. This interaction allows replacement of H3K4me3 marks by H3K27me3 and leads to the formation of a repressive chromatin state at *Pax7* promoter and to the repression of its expression [81]. Among TFs involved in differentiation, E2F is a family of eight proteins, parts of a complex including retinoblastoma-associated protein (RB), retinoblastoma-like protein 1 (RBL1) and 2 (RBL2) pocket proteins. Their function is to control the gene expression of proteins regulating the cell cycle and promoting differentiation in various tissues [82, 83]. RB, which was shown to bind HDAC1 for the maintenance of proliferation, can also regulate cell cycle exit through its interaction with E2F4. RB recruits HMT to promote H3K9me3 and H3K27me3 marks at the gene promoter of proteins promoting the cell cycle to decrease their expression, stop the cell cycle, and start differentiation. This repression is PRC1 and PRC2 dependent where the addition of H2AK119Ub1 and H3K27me3 marks establish a silent state [84]. This silent state could be maintained by the recruitment of dimerization partner, RB-like, E2F, and MuvB (dREAM) chromatin compaction complex to target genes through interactions between its L3MBTL1 subunit, E2F4 and HP1γ heterochromatin protein [85].

Induction of differentiation coincides with an increased expression of MYOD1. The increased *Myod1* expression is established through JMJD1c-driven demethylation of H3K9me3 marks in its promoter [36]. Once MYOD1 is expressed at high levels, a switch between the SNAI1/HDAC1/2 complex and MYOD1 occurs at E-box of muscle target genes to promote differentiation [60].

*Myog* expression is also essential for MuSCs to commit to differentiation (Fig. 2) and is regulated by a super enhancer upstream of the transcription start site [86]. The ability of TFs such as MYOD1, MEF2D, SIX4, and FOXO3 to create a transcriptional competent state at the myogenin promoter depends upon the combined activity of multiple epigenetic enzymes. One of the initial events in activating the *Myog* gene is the removal of the repressive H3K9me2 and H3K9me3 marks from the promoter by the action of the lysine demethylase JMJD2/KDM4A [87]. Moreover, focal adhesion kinase (FAK) helps achieve the open chromatin state by facilitating the departure of HDAC enzymes from the promoter. In this case, FAK binds the methyl-CpG-binding protein MBD2, where it induced phosphorylation of HDAC1 that breaks up the HDAC1/MBD2 interaction and dissociates it from the promoter [88]. Once the repressive marks are cleared, the promoter can then be modified to accumulate transcriptionally permissive marks. One of the first marks to appear is the dimethylation at arginine 8 in histone 3 (H3R8me2) within the promoter. This is achieved through the PRMT5 protein, a type II arginine methyltransferase. Once the H3R8me2 mark is in place, the epigenetic mark acts to allow a stable association of the chromatin remodeling complex SWI/SNF through recognition of modified histone tail by the Brg1 subunit of the complex. The association of SWI/SNF with the promoter then allows chromatin decompaction for RNA polymerase II to access the gene [89]. In addition, the histone methyltransferase SETD7 is targeted to the *Myog* promoter by MYOD1 to introduce the H3K4me1 mark. SETD7 is required for differentiation as silencing of SETD7 leads to a reduced number of myotubes and loss of expression of *Myog* [90–92]. The addition of H3K4me1 by SETD7 prevents the reintroduction of repressive H3K9me3 repressive marks by blocking Suv39h1 function [90–92]. Without the presence of HDAC1 at *Myog* promoter, the histone acetyltransferase P300/CAF leads to its enrichment of H3K9 and H3K14 acetylation and the expression of *Myog* [58]. The association of the histone acetyltransferase P300 with the *Myog* promoter is facilitated by chromatin-binding protein, NUPR1 (P8), which also recruits the RNA helicase DDX5 to the locus to promoter high levels of gene expression [93]. The introduction of H3K36me3 marks is also essential to high level expression of *Myog* as silencing of SETD2 blocked its expression and prevented myotube formation during differentiation [66]. Finally, histone exchanges within the nucleosome can alter the expression of the *Myog* gene. During the differentiation process, the subunit ZNHI1 (p18Hamlet) of SNF2 complex is phosphorylated by P38α which allows its recruitment to *Myog* promoter and allows the replacement of H2A histones by its less-stable variant H2A.Z (Fig. 2) [94].

For differentiation to proceed, cells must also begin to express functional genes that define the muscle lineage. Activation of these MYOD1 target genes requires the recruitment of SWI/SNF complex which is facilitated by the presence of histone 4 hyperacetylation that is recognized by the bromodomain of the transcription activator BRG1 (SMCA4), leading to chromatin decompaction and gene expression [95]. SWI/SNF recruitment is facilitated by the incorporation of the MYOD1-associated SMRD3 (BAF60C) subunit into the chromatin remodeling complex [96]. In addition, MyoD recruits the histone acetyl transferase P300, the JDP2, the AP1 (JUN) and RUNX1 TFs, and SETD7 HMT, leading to active histone modification marks H3K27Ac, H3K18ac, and H3K4me1 that target RNA polymerase II to the promoter region of muscle differentiation genes (Fig. 2) [97]. Interestingly, SETD7 methylates non-histone proteins such as the TF SRF. SRF acetylation promotes its binding to its serum response element at the muscle-specific gene *Acta1* to promote its expression. This regulation is necessary for differentiation and is revered by KDM2B [98]. These data suggest an indirect function of SETD7 in the regulation of differentiation. However, apart from *Acta1*, no other SRF targets have been identified in MuSCs so far.

While many repressive enzymes are removed from the MYOD1 target genes during differentiation, some complexes remain to permit repression of gene expression in response to a changing environment. An example of this is the *Myh4*, *Myog*, and *Ckm* genes which are marked by the Polycomb PRC2 mediated H3K27me3 modifications in proliferating MuSCs to repress their expression and to prevent differentiation. As differentiation initiates, a switch occurs between subunits in the PRC2 complex where the EZH2 subunit in proliferation (PRC2-EZH2) is replaced by a functionally inactive EZH1-containing PRC2 complex that lacks the EED subunit (PRC2-EZH1) [37, 99]. To permit gene expression during differentiation, removal of the H3K27me3 modifications is mediated by the H3K27 demethylase KDM6A (also known as UTX), a H3K27 demethylase that opens the chromatin to allow the expression of *Myog* and the entrance in differentiation [100, 101]. The stable association of PRC2-EZH1 complex that lacks methyltransferase activity helps to maintain the transcriptional permissive state [37, 99]. In response to cellular stresses such as muscle atrophy, the PRC2-EZH1 complex incorporates an EED subunit to form a functional HMT complex that can re-introduce H3K27me3 marks at these muscle genes to prevent their expression [37, 99].

As MYOG begins to push the differentiation program forward, additional epigenetic events will lead to the expression of terminal differentiation-related genes and fusion of differentiated myocytes into myotubes. TrxG complexes, containing a H3K4me3 methyl transferase ASH2L, are recruited by MEF2D to muscle-specific genes such as a muscle cytoplasmic enzyme, the muscle creatine kinase, where they mediate the addition of H3K4me3 marks that promote gene expression [102]. This recruitment is modulated through phosphorylation of MEF2D at threonine 308 and 315 by P38α MAPK. Interestingly, the function of a histone arginine methyltransferase Prmt5 is critical for early differentiation but is dispensable for late differentiation while the type I arginine methyltransferase, CARM1/PRMT4 is necessary for late differentiation as it deposits dimethylation of arginine 17 at histone 3 to permit gene activation [89, 103]. This reveals the refined roles of distinct epigenetic enzymes in ensuring the temporal expression of genes during muscle differentiation.

Finally, when fully differentiated, myocytes fuse thanks to Myomaker and Myomerger proteins to form multinucleated myofibers. While the epigenetic regulation of the Myomaker and Myomerger proteins has yet to be elucidated, the membrane protein CDON which positively regulates fusion was shown to be regulated by the Trithorax HMT ASH1L which deposits dimethylation of lysine 36 of histone 3 (H3K36me2) at the transcription start site to prevent Polycomb-mediated repression [104]. Interestingly, the absence of ASH1L provokes a diminution of fusion capacities of mouse and human myocytes in vitro without impairment of myosin heavy chain (MHC) protein expression. This suggests a direct control of fusion by a Trithorax complex and one can imagine that understanding of the regulation of fusion by epigenetic will emerge soon.

### DNA methylation modifications during differentiation

Several studies have revealed the important role of DNA methylation in regulating muscle differentiation. Indeed, during differentiation, the whole DNA methylation landscape was reported to decrease [105]. Several years ago, sodium arsenic treatment of C2C12 was reported to reduce differentiation capacities of the cells. This was correlated with increased DNA methylation of the CpG site of *Myog* promoter and a diminution of *Myog* expression [106]. Hypermethylation of *Myog* promoter in C2C12 cells decreases quickly after the induction of differentiation [73]. DNA methylation is known to repress TF binding. However, using a model of 293 T cells expressing luciferase reporter construct and different TFs, the binding of Sine oculis homeobox homolog 1 (SIX1) and MEF2A at *Myog* promoter was confirmed. Silencing SIX1 in C2C12 leads to increased methylation of *Myog* promoter, suggesting that SIX1 could have a role in the repression of the methylation [74, 107]. In the last few years, TET proteins have been shown to catalyze the conversion of DNA 5-methylcytosine into different oxidized forms, 5-hydroxymethylcytosine, 5-formylcytosine, and finally in 5-carboxylcytosine, demonstrating active demethylation capacities [108, 109]. Supporting the idea of a decrease in DNA methylation during differentiation, TET1 and TET2 expression are significatively increased in myoblasts after induction of differentiation. Interestingly, inhibition of TET2 but not TET1 by siRNA in C2C12 results in increased DNA methylation of *Myog*, *Myf6*/*Mrf4*, and *Mymk* (Myomaker) gene promoters. The increased DNA methylation at the promoter of these genes correlates with a decreased expression and an abrogation of C2C12 differentiation [110]. The presence of CpG methylation at *Myog* promoter is necessary for the binding of ZBT38 protein and the decreased expression of Myog. The diminution of methylation at *Myog* promoter may lead to ZBT38 removal and abrogation of its repression [75]. In addition to *Myog* promoter demethylation, a general diminution of DNA methylation at the CpG sites of *Myod1* promoter occurs after 3 days of differentiation in C2C12 cells [111]. DNA methylation changes during differentiation are not only attributed to demethylation of the promoter of genes required for myogenic differentiation. The addition of DNA methylation at CpG sites on the promoter of specific genes is also necessary to allow differentiation of myoblasts. In C2C12 cells, after 3 days of differentiation, an increase in DNA methylation of CpG sites of *Pax7* and *Myf5* promoters was shown. Interestingly, after 5 days of differentiation, the DNA methylation is still higher as compared with the proliferative state but slightly reduced as compared to day 3 of differentiation [111]. Genetic deletion of DNMT3A in mice resulted in fibrosis and reduction of cross-section area of the muscle after regeneration from an acute injury. The diminution of DNMT3A is correlated with a diminution of promoter DNA methylation and the expression of *Gdf5*, an important muscle gene. Interestingly, *Gdf5* increased expression does not change proliferation or differentiation capacities. However, a diminution of myofiber size, length, and nuclei number and a decreased expression of differentiation-related genes was reported, suggesting that DNA methylation of *Gdf5* promoter is necessary to avoid undesired muscle atrophy [112].

Modification of DNA methylation during differentiation is also correlated with histone modifications. In particular, heterochromatin protein HP1γ recognizes and binds H3K9me3 over the genome, interacts with DNMT1, and recruits HMTs [55, 113]. During this phenomenon, the protein level of HP1γ does not change, but its spatial localization does and is correlated with the presence of a methyl-CpG-binding protein, MECP2. These functions allow the maintenance of specific gene silencing by the addition of DNA methylation during differentiation and suggest interactions between DNA methylation and changes in histone PTMs [114].

## Changes of the muscle epigenome in muscle diseases

Aged and diseased muscles present an altered cell environment as compared with healthy adult muscles. Therefore, different cues trigger epigenome changes which will in turn alter the ability of MuSCs to maintain their functions.

Epigenome changes are well studied in the context of Duchenne muscular dystrophy (DMD). In this disease, loss of the dystrophin gene induces important muscle-degenerative phenotype in vivo. Constant myofiber damages are associated with an important inflammatory environment causing elevated levels of TNF, leading to diminution of the *Notch1* expression, an important mediator of activation and proliferation of MuSCs [115, 116]. The repression of Notch1 protein is due to an increase of DNA methylation of *Notch1* led by EZH2-dependent recruitment of DNMT3b [117]. Diminution of Notch signaling could impair MuSC maintenance in the quiescence state by modification of DNA methylation landscape. The absence of a functional dystrophin complex also conducts to an alteration of its related nitric oxide (NO) pathway. The histone acetyl transferase CBP/P300 was shown to be downregulated in the zebrafish model of DMD while its overexpression in embryonic development rescues the phenotype [118]. This suggests the importance of epigenetic factors in the ability of the muscle to resist damages in dystrophy. Moreover, an abnormal pattern of histone modifications is present in proliferative myoblasts of human DMD and the *mdx* mouse model of DMD. This global change in histone PTMs is characterized by an increased level of H3K14 and H3K9 acetylation, augmentation of H3K79me2 marks, and an increase of phosphorylation of H3 serine 10 [119]. The decreased expression of BMI1, a subunit of PRC1 complex, was revealed in human DMD myoblasts as compared with healthy myoblasts. Interestingly, BMI1 overexpression reduces oxidative stress and DNA damages and increases ATP production in DMD myoblasts [120]. It is interesting to note that the absence of BMI1 expression causes dysregulation of p16^INK4a^ and early senescence of MuSCs [26]. Thus, epigenetic changes in MuSCs from DMD patients lead to functional exhaustion of the stem cell pool.

Another example of a myopathy in which epigenome is altered is Emery-Dreifuss muscular dystrophy. In this disease, mutations in *Lamin A/C* gene, encoding for the nuclear envelop protein Lamin A/C, alter chromatin condensation in MuSCs. Furthermore, the altered interactions between chromatin and nuclear lamina cause deregulation of the H3K27me3-mediating Polycomb complex in MuSCs [121]. This change in Polycomb complex position leads to a diminution of self-renewal and exhaustion of MuSCs [121].

Age-related inflammation also creates an altered muscle environment that changes MuSC epigenome. Among these changes, DNA methylation marks are well known to change with age and environmental exposure, suggesting a potential modification of MuSC epigenome over time [122]. In human, a comparison of DNA methylation revealed hypomethylation near the 5′ region and hypermethylation at the middle and 3′ gene regions of genes in old (68–89 years old) versus young (18–27 years old) human skeletal muscle [123]. Moreover, hypermethylation of intragenic regions of genes involved in motor neuron junctions and myofibers formation was shown [123]. A correlation was found between gene underexpression and hypermethylation of intragenic, 5′ and at transcription start regions. Opposingly, upregulation of genes is correlated with hypomethylation of intragenic, 5′ and transcription start regions [123]. Such gene deregulation can be correlated to the loss of motor units and denervation observed in muscle in sarcopenia. Gene deregulation related to the modification of the DNA methylation during aging is observed in muscles of older subjects [123, 124]. In humans, the increased DNA methylation was observed at the promoter of genes coding for components of mitochondrial respiratory chain, COX7A1 and NDUFB6, which is related to decrease expression of the genes in the elderly. These alterations exhibit a direct effect of DNA methylation alteration with age [125, 126]. A study performed in MuSCs isolated from young and old mice suggested that a change of DNA methylation during aging is a stochastic event. These events occur at gene promoters and drive inter-variability gene expression between myoblasts [127]. An important decline of muscle regenerative functions is also observed. Studies performed on young (2–6 months), old (18–24 months), and geriatric (26–36 months) mice exposed loss of MuSC functions with aging, even when the cells were transplanted in young muscle, suggesting intrinsic alterations of MuSCs. These changes are caused by deregulation of p16^INK4a^ by PRC1 subunit BMI1, which leads geriatric MuSCs to a switch in a pre-senescence state (Fig. 3) [26, 128]. This conversion of MuSCs to a senescent state has the unwanted consequence of reducing the number of functional stem cells available for repair of muscle wasting. Thus, changes in the muscle environment in both aging and disease lead to functional exhaustion of MuSCs.

## Altered cell fate in muscle disease and aging

As discussed above, the identity of a cell is defined by tissue-specific gene expression programs that are determined through epigenetic mechanisms. As such, an altered cell environment may alter MuSC fate through epigenetic mechanisms. Cell plasticity due to changes in the epigenome was established in experiments showing that treatment of fibroblast cell line with the DNA methylation inhibitor 5-azacytidine triggers the activation of MYOD1 and the formation of multinucleated myotubes [38, 129, 130]. Cell plasticity in response to specific stimuli has since been more extensively studied in terms of pluripotency where fibroblasts were reprogrammed to the embryonic state by the formation of induced pluripotent stem cells [131–133]. While the induced pluripotent stem cells were first derived using a combination of pluripotency TFs, later studies showed that the lineage conversion mechanisms can be similarly driven by exposure to a variety of small molecule inhibitors [134, 135]. As such, it should not be surprising that altered muscle environment in myopathies and aging might give rise to alternate MuSC fates.

In recent years, evidence started to accumulate that MuSCs can give rise to fibroblasts in DMD and aging. An extracellular component of the aged muscle was shown to induce a lineage conversion of MuSCs towards the fibroblast lineage [136]. Lineage conversion in response to environmental cues was confirmed in lineage tracing experiment using PAX7^CreER^;R26R^YFP^ bred with *mdx* mouse, in which 7 to 20% of MuSCs acquire a fibroblast phenotype [137]. Additionally, *mdx* mouse muscle shows elevated TGFβ and Wnt signaling, which induces the myogenic to fibrogenic conversion. Indeed, Wnt signaling controls TGFβ2 expression to induce fibrogenic conversion [137, 138]. Accordingly, inhibition of WNT-TGFβ2 signaling prevents the lineage conversion and reduces the expression of fibrogenic genes [137–139]. Mechanisms sustaining such changes of MuSC fate due to altered environment are still unknown and need to be investigated, as well as their evidence in human since fate conversion was demonstrated in the mouse.

Finally, similar to the problem discussed in for Emery-Dreifuss muscular dystrophy, a loss of functional MuSCs due to p16^INK4a^ expression has also been shown in sarcopenia. In this case, downregulation of BMI1 leads to the displacement of PRC1 complex from the promoter of p16^INK4a^ in geriatric MuSCs. In the absence of PRC1, inappropriate removal of PRC1 complex decreases the presence of its repressive marks H2Aub, increases p16^INK4a^ expression, leading MuSCs to senescence [26]. While similar approaches of dead Cas9 (dCas9)-Cbx4 to repress the p16^ink4a^ gene would also be effective, the use of gene therapy to prevent aging pushes the ethical boundaries. Instead, small molecule treatments to maintain Bmi1 expression in aged MuSCs would seem to be a more appropriate means to ensure PRC1 mediated repression of the p16^INK4a^ promoter to reduce senescence in aged MuSCs.

## Conclusion

The study of mechanisms allowing total regeneration of muscles by MuSCs had started a few decades ago. After the discovery of MRFs, the understanding of epigenetic mechanisms added new insights in the transition of each step of myogenesis. Separately, DNA methylation and histone post-transcriptional regulation have been well studied in muscle, although many unanswered aspects still remain. Recent studies highlighted the importance of considering that these regulations are changing in a spacio/temporal manner. Because of its complexity, tight dysregulation of any of these mechanisms can lead to an abnormal myogenic program and incapacity to correctly regenerate the muscle. Alterations of the MuSC epigenome encountered during aging or muscle pathology conduct gene dysregulations and to the diminution of the capacity of MuSCs to regenerate. Recently, modifications of these regulations were reported to alter the maintenance of a healthy MuSC fate in mouse models [137, 139]. These modifications are not well understood yet. Possibly, the important advances in technologies to analyze epigenomes of a small number of cells will allow the discovering of the mechanisms leading to abnormal cell fate decisions of MuSCs.

## Data Availability

Not applicable.

## Abbreviations

AAV: Adeno-associated virus
CER: Core enhancer region
dCas9: Dead Cas9
DMD: Duchenne muscular dystrophy
DNMT: DNA methyltransferase
dREAM: Dimerization partner, RB-like, E2F, and MuvB
DRR: Distal regulatory region
FAK: Focal adhesion kinase
H2AUb: Monoubiquitination of lysine 119 of histone 2A
H3K20me1: Monomethylation of lysine 20 of histone 3
H3K27Ac: Acetylation of lysine 27 of histone 3
H3K27me3: Trimethylation of lysine 9 of histone 3
H3K36me2: Dimethylation of lysine 36 of histone 3
H3K4me3: Trimethylation of the lysine 4 of histone 3
H3K9Ac: Acetylation of lysine 9 of histone 3
H3K9me3: Trimethylation of lysine 9 of histone 3
H3R8me2: Dimethylation of arginine 8 of histone 3
H4K20Ac: Acetylation of lysine 20 of histone 4
H4K20me3: Trimethylation of lysine 20 of histone 4
HDAC: Histone deacetylase
HMT: Histone methyltransferase
MAPK: Mitogen-activated protein kinase
MCK: Muscle creatine kinase
MeCP2: Methyl-CpG-binding protein 2
MHC: Myosine heavy chain
MRF: Myogenic regulatory factor
MuSC: Muscle stem cell
MYOG: Myogenin
NO: Nitric oxide
PCAF: P300/CBP-associated factor
PcG: Polycomb group
PRC: Polycomb repressive complex
PTM: Post-translational modification
SWI/SNF: Switch/sucrose nonfermentable chromatin remodeling complex
TET: Ten-eleven translocation
TF: Transcription factor
TrxG: Trithorax group

## References

[1] Ou HD, Phan S, Deerinck TJ, Thor A, Ellisman MH, O’Shea CC (2017). ChromEMT: Visualizing 3D chromatin structure and compaction in interphase and mitotic cells. Science.

[2] Bintu B, Mateo LJ, Su J-H, Sinnott-Armstrong NA, Parker M, Kinrot S (2018). Super-resolution chromatin tracing reveals domains and cooperative interactions in single cells. Science.

[3] Mohandas T, Sparkes R, Shapiro L (1981). Reactivation of an inactive human X chromosome: evidence for X inactivation by DNA methylation. Science..

[4] Rice JC, Allis CD (2001). Histone methylation versus histone acetylation: new insights into epigenetic regulation. Curr Opin Cell Biol..

[5] Schuettengruber B, Martinez A-M, Iovino N, Cavalli G (2011). Trithorax group proteins: switching genes on and keeping them active. Nat Rev Mol Cell Biol..

[6] Zhang CL, McKinsey TA, Olson EN (2002). Association of class II histone deacetylases with heterochromatin protein 1: potential role for histone methylation in control of muscle differentiation. Mol Cell Biol..

[7] Heintzman ND, Stuart RK, Hon G, Fu Y, Ching CW, Hawkins RD (2007). Distinct and predictive chromatin signatures of transcriptional promoters and enhancers in the human genome. Nat Genet..

[8] Herz H-M, Mohan M, Garruss AS, Liang K, Takahashi Y –h, Mickey K (2012). Enhancer-associated H3K4 monomethylation by Trithorax-related, the Drosophila homolog of mammalian Mll3/Mll4. Genes Dev.

[9] Mauro A (1961). Satellite cell of skeletal muscle fibers. J Biophys Biochem Cytol..

[10] Aziz A, Liu Q-C, Dilworth FJ (2010). Regulating a master regulator: establishing tissue-specific gene expression in skeletal muscle. Epigenetics..

[11] Segalés J, Perdiguero E, Muñoz-Cánoves P (2015). Epigenetic control of adult skeletal muscle stem cell functions. FEBS J..

[12] Singh K, Dilworth FJ (2013). Differential modulation of cell cycle progression distinguishes members of the myogenic regulatory factor family of transcription factors. FEBS J..

[13] García-Prat L, Perdiguero E, Alonso-Martín S, Dell’Orso S, Ravichandran S, Brooks SR, et al. FoxO maintains a genuine muscle stem-cell quiescent state until geriatric age. Nat Cell Biol. 2020. Available from: http://www.nature.com/articles/s41556-020-00593-7. 10.1038/s41556-020-00593-7 33106654

[14] Beauchamp JR, Heslop L, Yu DS, Tajbakhsh S, Kelly RG, Wernig A (2000). Expression of CD34 and Myf5 defines the majority of quiescent adult skeletal muscle satellite cells. J Cell Biol..

[15] Crist CG, Montarras D, Buckingham M (2012). Muscle satellite cells are primed for myogenesis but maintain quiescence with sequestration of Myf5 mRNA targeted by microRNA-31 in mRNP granules. Cell Stem Cell..

[16] Yue L, Wan R, Luan S, Zeng W, Cheung TH. Dek modulates global intron retention during muscle stem cells quiescence exit. Dev Cell. 2020;53:661–76. 10.1016/j.devcel.2020.05.006 32502396

[17] Kuang S, Kuroda K, Le Grand F, Rudnicki MA (2007). Asymmetric self-renewal and commitment of satellite stem cells in muscle. Cell..

[18] Yablonka-Reuveni Z, Rudnicki MA, Rivera AJ, Primig M, Anderson JE, Natanson P (1999). The transition from proliferation to differentiation is delayed in satellite cells from mice lacking MyoD. Dev Biol..

[19] Hinterberger TJ, Sassoon DA, Rhodes SJ, Konieczny SF (1991). Expression of the muscle regulatory factor MRF4 during somite and skeletal myofiber development. Dev Biol..

[20] Lazure F, Blackburn DM, Corchado AH, Sahinyan K, Karam N, Sharanek A, et al. Myf6/MRF4 is a myogenic niche regulator required for the maintenance of the muscle stem cell pool. EMBO Rep. 2020. Available from: https://onlinelibrary.wiley.com/doi/10.15252/embr.201949499. 10.15252/embr.201949499 PMC7726801 33047485

[21] Zhu Z, Boone MJ (1997). MRF4 can substitute for myogenin during early stages of myogenesis. Dev Dyn..

[22] Machado L, Esteves de Lima J, Fabre O, Proux C, Legendre R, Szegedi A (2017). In situ fixation redefines quiescence and early activation of skeletal muscle stem cells. Cell Rep..

[23] van Velthoven CTJ, de Morree A, Egner IM, Brett JO, Rando TA (2017). Transcriptional profiling of quiescent muscle stem cells in vivo. Cell Rep..

[24] Liu L, Cheung TH, Charville GW, Hurgo BMC, Leavitt T, Shih J (2013). Chromatin modifications as determinants of muscle stem cell quiescence and chronological aging. Cell Rep..

[25] Liu L, Cheung TH, Charville GW, Rando TA (2015). Isolation of skeletal muscle stem cells by fluorescence-activated cell sorting. Nat Protoc..

[26] Sousa-Victor P, Gutarra S, García-Prat L, Rodriguez-Ubreva J, Ortet L, Ruiz-Bonilla V (2014). Geriatric muscle stem cells switch reversible quiescence into senescence. Nature..

[27] Gopinath SD, Webb AE, Brunet A, Rando TA (2014). FOXO3 Promotes quiescence in adult muscle stem cells during the process of self-renewal. Stem Cell Rep..

[28] Bjornson CRR, Cheung TH, Liu L, Tripathi PV, Steeper KM, Rando TA (2012). Notch signaling is necessary to maintain quiescence in adult muscle stem cells. STEM CELLS..

[29] Boonsanay V, Zhang T, Georgieva A, Kostin S, Qi H, Yuan X (2016). Regulation of skeletal muscle stem cell quiescence by Suv4-20 h1-dependent facultative heterochromatin formation. Cell Stem Cell..

[30] Li Y, Dilworth FJ (2016). compacting chromatin to ensure muscle satellite cell quiescence. Cell Stem Cell..

[31] Addicks GC, Brun CE, Sincennes M-C, Saber J, Porter CJ, Francis Stewart A (2019). MLL1 is required for PAX7 expression and satellite cell self-renewal in mice. Nat Commun..

[32] Cao R, Tsukada Y, Zhang Y (2005). Role of Bmi-1 and Ring1A in H2A Ubiquitylation and Hox gene silencing. Mol Cell..

[33] Wang H, Wang L, Erdjument-Bromage H, Vidal M, Tempst P, Jones RS (2004). Role of histone H2A ubiquitination in Polycomb silencing. Nature..

[34] Agherbi H, Gaussmann-Wenger A, Verthuy C, Chasson L, Serrano M, Djabali M (2009). Polycomb mediated epigenetic silencing and replication timing at the INK4a/ARF locus during senescence. Blagosklonny MV, editor. PLoS ONE.

[35] Juan AH, Derfoul A, Feng X, Ryall JG, Dell’Orso S, Pasut A (2011). Polycomb EZH2 controls self-renewal and safeguards the transcriptional identity of skeletal muscle stem cells. Genes Dev..

[36] Luo D, de Morree A, Boutet S, Quach N, Natu V, Rustagi A (2017). Deltex2 represses MyoD expression and inhibits myogenic differentiation by acting as a negative regulator of Jmjd1c. Proc Natl Acad Sci U S A..

[37] Caretti G (2004). The Polycomb Ezh2 methyltransferase regulates muscle gene expression and skeletal muscle differentiation. Genes Dev..

[38] Jones PA, Wolkowicz MJ, Rideout WM, Gonzales FA, Marziasz CM, Coetzee GA (1990). De novo methylation of the MyoD1 CpG island during the establishment of immortal cell lines. Proc Natl Acad Sci..

[39] Diede SJ, Yao Z, Keyes CC, Tyler AE, Dey J, Hackett CS (2013). Fundamental differences in promoter CpG island DNA hypermethylation between human cancer and genetically engineered mouse models of cancer. Epigenetics..

[40] McKinnell IW, Ishibashi J, Le Grand F, Punch VGJ, Addicks GC, Greenblatt JF (2008). Pax7 activates myogenic genes by recruitment of a histone methyltransferase complex. Nat Cell Biol..

[41] Diao Y, Guo X, Li Y, Sun K, Lu L, Jiang L (2012). Pax3/7BP Is a Pax7- and Pax3-binding protein that regulates the proliferation of muscle precursor cells by an epigenetic mechanism. Cell Stem Cell..

[42] Kawabe Y, Wang YX, McKinnell IW, Bedford MT, Rudnicki MA (2012). Carm1 Regulates Pax7 transcriptional activity through MLL1/2 recruitment during asymmetric satellite stem cell divisions. Cell Stem Cell..

[43] Cai S, Zhu Q, Guo C, Yuan R, Zhang X, Nie Y, et al. MLL1 promotes myogenesis by epigenetically regulating *Myf5*. Cell Prolif. 2020;53 Available from: https://onlinelibrary.wiley.com/doi/abs/10.1111/cpr.12744. [cited 2020 Mar 10]. 10.1111/cpr.12744 PMC7046306 31840352

[44] Padilla-Benavides T, Nasipak BT, Imbalzano AN (2015). Brg1 Controls the expression of *Pax7* to promote viability and proliferation of mouse primary myoblasts: primary myoblasts require Brg1. J Cell Physiol..

[45] Padilla-Benavides T, Nasipak BT, Paskavitz AL, Haokip DT, Schnabl JM, Nickerson JA (2017). Casein kinase 2-mediated phosphorylation of Brahma-related gene 1 controls myoblast proliferation and contributes to SWI/SNF complex composition. J Biol Chem..

[46] Lilja KC, Zhang N, Magli A, Gunduz V, Bowman CJ, Arpke RW (2017). Pax7 remodels the chromatin landscape in skeletal muscle stem cells. PloS One..

[47] Lee H (2004). Msx1 Cooperates with histone H1b for inhibition of transcription and myogenesis. Science..

[48] Puri PL, Iezzi S, Stiegler P, Chen TT, Schiltz RL, Muscat GE (2001). Class I histone deacetylases sequentially interact with MyoD and pRb during skeletal myogenesis. Mol Cell..

[49] Bröhl D, Vasyutina E, Czajkowski MT, Griger J, Rassek C, Rahn H-P (2012). Colonization of the satellite cell niche by skeletal muscle progenitor cells depends on Notch signals. Dev Cell..

[50] Lahmann I, Bröhl D, Zyrianova T, Isomura A, Czajkowski MT, Kapoor V (2019). Oscillations of MyoD and Hes1 proteins regulate the maintenance of activated muscle stem cells. Genes Dev..

[51] Singh K, Cassano M, Planet E, Sebastian S, Jang SM, Sohi G (2015). A KAP1 phosphorylation switch controls MyoD function during skeletal muscle differentiation. Genes Dev..

[52] Ling BMT, Bharathy N, Chung T-K, Kok WK, Li S, Tan YH (2012). Lysine methyltransferase G9a methylates the transcription factor MyoD and regulates skeletal muscle differentiation. Proc Natl Acad Sci..

[53] Dilworth FJ, Seaver KJ, Fishburn AL, Htet SL, Tapscott SJ (2004). In vitro transcription system delineates the distinct roles of the coactivators pCAF and p300 during MyoD/E47-dependent transactivation. Proc Natl Acad Sci..

[54] Choi J, Jang H, Kim H, Kim S-T, Cho E-J, Youn H-D (2010). Histone demethylase LSD1 is required to induce skeletal muscle differentiation by regulating myogenic factors. Biochem Biophys Res Commun..

[55] Ait-Si-Ali S, Guasconi V, Fritsch L, Yahi H, Sekhri R, Naguibneva I (2004). A Suv39h-dependent mechanism for silencing S-phase genes in differentiating but not in cycling cells. EMBO J..

[56] Fulco M, Schiltz RL, Iezzi S, King MT, Zhao P, Kashiwaya Y (2003). Sir2 regulates skeletal muscle differentiation as a potential sensor of the redox state. Mol Cell..

[57] Mal AK (2006). Histone methyltransferase Suv39h1 represses MyoD-stimulated myogenic differentiation. EMBO J..

[58] Mal A, Harter ML (2003). MyoD is functionally linked to the silencing of a muscle-specific regulatory gene prior to skeletal myogenesis. Proc Natl Acad Sci..

[59] Gillespie MA, Le Grand F, Scimè A, Kuang S, von Maltzahn J, Seale V (2009). p38-γ–dependent gene silencing restricts entry into the myogenic differentiation program. J Cell Biol..

[60] Soleimani VD, Yin H, Jahani-Asl A, Ming H, Kockx CEM, van Ijcken WFJ (2012). Snail regulates MyoD binding-site occupancy to direct enhancer switching and differentiation-specific transcription in myogenesis. Mol Cell..

[61] Takahashi Y, Rayman JB, Dynlacht BD (2000). Analysis of promoter binding by the E2F and pRB families in vivo: distinct E2F proteins mediate activation and repression. Genes Dev..

[62] Taubert S, Gorrini C, Frank SR, Parisi T, Fuchs M, Chan H-M (2004). E2F-dependent histone acetylation and recruitment of the Tip60 acetyltransferase complex to chromatin in late G1. Mol Cell Biol..

[63] Rao VK, Ow JR, Shankar SR, Bharathy N, Manikandan J, Wang Y (2016). G9a promotes proliferation and inhibits cell cycle exit during myogenic differentiation. Nucleic Acids Res..

[64] Nightingale KP, Gendreizig S, White DA, Bradbury C, Hollfelder F, Turner BM (2007). Cross-talk between histone modifications in response to histone deacetylase inhibitors: MLL4 links histone H3 acetylation and histone h3k4 methylation. J Biol Chem..

[65] Sebastian S, Sreenivas P, Sambasivan R, Cheedipudi S, Kandalla P, Pavlath GK (2009). MLL5, a trithorax homolog, indirectly regulates H3K4 methylation, represses cyclin A2 expression, and promotes myogenic differentiation. Proc Natl Acad Sci..

[66] Yi X, Tao Y, Lin X, Dai Y, Yang T, Yue X (1864). Histone methyltransferase Setd2 is critical for the proliferation and differentiation of myoblasts. Biochim Biophys Acta Mol Cell Res..

[67] Pietrocola F, Galluzzi L, Bravo-San Pedro JM, Madeo F, Kroemer G (2015). Acetyl Coenzyme A: a central metabolite and second messenger. Cell Metab..

[68] Yucel N, Wang YX, Mai T, Porpiglia E, Lund PJ, Markov G (2019). Glucose metabolism drives histone acetylation landscape transitions that dictate muscle stem cell function. Cell Rep.

[69] Ryall JG, Dell’Orso S, Derfoul A, Juan A, Zare H, Feng X (2015). The NAD(+)-dependent SIRT1 deacetylase translates a metabolic switch into regulatory epigenetics in skeletal muscle stem cells. Cell Stem Cell..

[70] Naito M, Mori M, Inagawa M, Miyata K, Hashimoto N, Tanaka S (2016). Dnmt3a regulates proliferation of muscle satellite cells via p57Kip2. PLoS Genet..

[71] Yokoyama S, Ito Y, Ueno-Kudoh H, Shimizu H, Uchibe K, Albini S (2009). A systems approach reveals that the myogenesis genome network is regulated by the transcriptional repressor RP58. Dev Cell..

[72] Fuks F (2001). Dnmt3a binds deacetylases and is recruited by a sequence-specific repressor to silence transcription. EMBO J..

[73] Fuso A, Ferraguti G, Grandoni F, Ruggeri R, Scarpa S, Strom R (2010). Early demethylation of non-CpG, CpC-rich, elements in the myogenin 5’-flanking region: a priming effect on the spreading of active demethylation?. Cell Cycle..

[74] Palacios D, Summerbell D, Rigby PWJ, Boyes J (2010). Interplay between DNA methylation and transcription factor availability: implications for developmental activation of the mouse myogenin gene. Mol Cell Biol..

[75] Oikawa Y, Omori R, Nishii T, Ishida Y, Kawaichi M, Matsuda E (2011). The methyl-CpG-binding protein CIBZ suppresses myogenic differentiation by directly inhibiting myogenin expression. Cell Res..

[76] Skapek SX, Rhee J, Kim PS, Novitch BG, Lassar AB (1996). Cyclin-mediated inhibition of muscle gene expression via a mechanism that is independent of pRB hyperphosphorylation. Mol Cell Biol..

[77] Zhang P, Wong C, Liu D, Finegold M, Harper JW, Elledge SJ (1999). p21(CIP1) and p57(KIP2) control muscle differentiation at the myogenin step. Genes Dev..

[78] Berkes CA, Tapscott SJ (2005). MyoD and the transcriptional control of myogenesis. Semin Cell Dev Biol..

[79] Bhanu NV, Sidoli S, Yuan Z-F, Molden RC, Garcia BA (2019). Regulation of proline-directed kinases and the trans-histone code H3K9me3/H4K20me3 during human myogenesis. J Biol Chem..

[80] Asp P, Blum R, Vethantham V, Parisi F, Micsinai M, Cheng J (2011). Genome-wide remodeling of the epigenetic landscape during myogenic differentiation. Proc Natl Acad Sci..

[81] Palacios D, Mozzetta C, Consalvi S, Caretti G, Saccone V, Proserpio V (2010). TNF/p38α/polycomb signaling to Pax7 locus in satellite cells links inflammation to the epigenetic control of muscle regeneration. Cell Stem Cell..

[82] Balciunaite E, Spektor A, Lents NH, Cam H, te Riele H, Scime A (2005). Pocket protein complexes are recruited to distinct targets in quiescent and proliferating cells. Mol Cell Biol..

[83] Dimova DK (2003). Cell cycle-dependent and cell cycle-independent control of transcription by the Drosophila E2F/RB pathway. Genes Dev..

[84] Schwartz YB, Pirrotta V (2007). Polycomb silencing mechanisms and the management of genomic programmes. Nat Rev Genet..

[85] Trojer P, Li G, Sims RJ, Vaquero A, Kalakonda N, Boccuni P (2007). L3MBTL1, a histone-methylation-dependent chromatin lock. Cell..

[86] Peng XL, So KK, He L, Zhao Y, Zhou J, Li Y (2017). MyoD- and FoxO3-mediated hotspot interaction orchestrates super-enhancer activity during myogenic differentiation. Nucleic Acids Res..

[87] Verrier L, Escaffit F, Chailleux C, Trouche D, Vandromme M (2011). A new isoform of the histone demethylase JMJD2A/KDM4A is required for skeletal muscle differentiation. Cox GA, editor. PLoS Genet.

[88] Luo S-W, Zhang C, Zhang B, Kim C-H, Qiu Y-Z, Du Q-S (2009). Regulation of heterochromatin remodelling and myogenin expression during muscle differentiation by FAK interaction with MBD2. EMBO J..

[89] Dacwag CS, Ohkawa Y, Pal S, Sif S, Imbalzano AN (2007). The protein arginine methyltransferase Prmt5 is required for myogenesis because it facilitates ATP-dependent chromatin remodeling. Mol Cell Biol..

[90] Nishioka K (2002). Set9, a novel histone H3 methyltransferase that facilitates transcription by precluding histone tail modifications required for heterochromatin formation. Genes Dev..

[91] Wang H, Cao R, Xia L, Erdjument-Bromage H, Borchers C, Tempst P (2001). Purification and functional characterization of a histone H3-lysine 4-specific methyltransferase. Mol Cell..

[92] Tao Y, Neppl RL, Huang Z-P, Chen J, Tang R-H, Cao R (2011). The histone methyltransferase Set7/9 promotes myoblast differentiation and myofibril assembly. J Cell Biol..

[93] Sambasivan R, Cheedipudi S, Pasupuleti N, Saleh A, Pavlath GK, Dhawan J (2009). The small chromatin-binding protein p8 coordinates the association of anti-proliferative and pro-myogenic proteins at the myogenin promoter. J Cell Sci..

[94] Cuadrado A, Corrado N, Perdiguero E, Lafarga V, Muñoz-Canoves P, Nebreda AR (2010). Essential role of p18Hamlet/SRCAP-mediated histone H2A.Z chromatin incorporation in muscle differentiation. EMBO J.

[95] de la Serna IL, Ohkawa Y, Berkes CA, Bergstrom DA, Dacwag CS, Tapscott SJ (2005). MyoD targets chromatin remodeling complexes to the myogenin locus prior to forming a stable DNA-bound complex. Mol Cell Biol..

[96] Forcales SV, Albini S, Giordani L, Malecova B, Cignolo L, Chernov A (2012). Signal-dependent incorporation of MyoD-BAF60c into Brg1-based SWI/SNF chromatin-remodelling complex: BAF60c-MyoD marks chromatin for SWI/SNF recruitment. EMBO J..

[97] Blum R, Dynlacht BD (2013). The role of MyoD1 and histone modifications in the activation of muscle enhancers. Epigenetics..

[98] Joung H, Kang J-Y, Kim J-Y, Kwon D-H, Jeong A, Min H-K (2020). SRF is a non-histone methylation target of KDM2B and SET7 in the regulation of myogenesis. bioRxiv.

[99] Stojic L, Jasencakova Z, Prezioso C, Stützer A, Bodega B, Pasini D (2011). Chromatin regulated interchange between polycomb repressive complex 2 (PRC2)-Ezh2 and PRC2-Ezh1 complexes controls myogenin activation in skeletal muscle cells. Epigenetics Chromatin..

[100] Faralli H, Wang C, Nakka K, Benyoucef A, Sebastian S, Zhuang L (2016). UTX demethylase activity is required for satellite cell–mediated muscle regeneration. J Clin Invest..

[101] Seenundun S, Rampalli S, Liu Q-C, Aziz A, Palii C, Hong S (2010). UTX mediates demethylation of H3K27me3 at muscle-specific genes during myogenesis. EMBO J..

[102] Rampalli S, Li L, Mak E, Ge K, Brand M, Tapscott SJ (2007). p38 MAPK signaling regulates recruitment of Ash2L-containing methyltransferase complexes to specific genes during differentiation. Nat Struct Mol Biol..

[103] Dacwag CS, Bedford MT, Sif S, Imbalzano AN (2009). Distinct protein arginine methyltransferases promote ATP-dependent chromatin remodeling function at different stages of skeletal muscle differentiation. Mol Cell Biol..

[104] Castiglioni I, Caccia R, Garcia-Manteiga JM, Ferri G, Caretti G, Molineris I (2018). The Trithorax protein Ash1L promotes myoblast fusion by activating Cdon expression. Nat Commun..

[105] Tsumagari K, Baribault C, Terragni J, Varley KE, Gertz J, Pradhan S (2013). Early de novo DNA methylation and prolonged demethylation in the muscle lineage. Epigenetics..

[106] Steffens AA, Hong G-M, Bain LJ (2011). Sodium arsenite delays the differentiation of C2C12 mouse myoblast cells and alters methylation patterns on the transcription factor myogenin. Toxicol Appl Pharmacol..

[107] Liu Y, Chu A, Chakroun I, Islam U, Blais A (2010). Cooperation between myogenic regulatory factors and SIX family transcription factors is important for myoblast differentiation. Nucleic Acids Res..

[108] Pfaffeneder T, Hackner B, Truß M, Münzel M, Müller M, Deiml CA (2011). The discovery of 5-formylcytosine in embryonic stem cell DNA. Angew Chem Int Ed..

[109] Tahiliani M, Koh KP, Shen Y, Pastor WA, Bandukwala H, Brudno Y (2009). Conversion of 5-methylcytosine to 5-hydroxymethylcytosine in mammalian DNA by MLL Partner TET1. Science..

[110] Zhong X, Wang Q-Q, Li J-W, Zhang Y-M, An X-R, Hou J (2017). Ten-eleven translocation-2 (Tet2) is involved in myogenic differentiation of skeletal myoblast cells in vitro. Sci Rep..

[111] Chao Z, Zheng X-L, Sun R-P, Liu H-L, Huang L-L, Cao Z-X (2016). Characterization of the methylation status of Pax7 and myogenic regulator factors in cell myogenic differentiation. Asian-Australas J Anim Sci..

[112] Hatazawa Y, Ono Y, Hirose Y, Kanai S, Fujii NL, Machida S (2018). Reduced Dnmt3a increases Gdf5 expression with suppressed satellite cell differentiation and impaired skeletal muscle regeneration. FASEB J Off Publ Fed Am Soc Exp Biol..

[113] Smallwood A, Esteve P-O, Pradhan S, Carey M (2007). Functional cooperation between HP1 and DNMT1 mediates gene silencing. Genes Dev..

[114] Agarwal N, Hardt T, Brero A, Nowak D, Rothbauer U, Becker A (2007). MeCP2 interacts with HP1 and modulates its heterochromatin association during myogenic differentiation. Nucleic Acids Res..

[115] Tidball JG (2005). Inflammatory processes in muscle injury and repair. Am J Physiol-Regul Integr Comp Physiol..

[116] Porter JD (2002). A chronic inflammatory response dominates the skeletal muscle molecular signature in dystrophin-deficient mdx mice. Hum Mol Genet..

[117] Acharyya S, Sharma SM, Cheng AS, Ladner KJ, He W, Kline W (2010). TNF inhibits Notch-1 in skeletal muscle cells by Ezh2 and DNA methylation mediated repression: implications in Duchenne muscular dystrophy. Bryk M, editor. PLoS ONE.

[118] Bajanca F, Vandel L. Epigenetic regulators modulate muscle damage in Duchenne muscular dystrophy model. PLoS Curr. 2017. Available from: http://currents.plos.org/md/article/epigenetic-regulators-modulate-muscle-damage-in-duchenne-muscular-dystrophy-model/. 10.1371/currents.md.f1e2379fa632f8135577333dd92ca83b PMC5774996 29399383

[119] Colussi C, Gurtner A, Rosati J, Illi B, Ragone G, Piaggio G (2009). Nitric oxide deficiency determines global chromatin changes in Duchenne muscular dystrophy. FASEB J..

[120] Dibenedetto S, Niklison-Chirou M, Cabrera CP, Ellis M, Robson LG, Knopp P (2017). Enhanced energetic state and protection from oxidative stress in human myoblasts overexpressing BMI1. Stem Cell Rep..

[121] Bianchi A, Mozzetta C, Pegoli G, Lucini F, Valsoni S, Rosti V (2020). Dysfunctional polycomb transcriptional repression contributes to lamin A/C–dependent muscular dystrophy. J Clin Invest..

[122] Hannum G, Guinney J, Zhao L, Zhang L, Hughes G, Sadda S (2013). Genome-wide methylation profiles reveal quantitative views of human aging rates. Mol Cell..

[123] Zykovich A, Hubbard A, Flynn JM, Tarnopolsky M, Fraga MF, Kerksick C (2014). Genome-wide DNA methylation changes with age in disease-free human skeletal muscle. Aging Cell..

[124] Parker MH. The altered fate of aging satellite cells is determined by signaling and epigenetic changes. Front Genet. 2015;6. Available from: http://journal.frontiersin.org/Article/10.3389/fgene.2015.00059/abstract. 10.3389/fgene.2015.00059 PMC4335604 25750654

[125] Ling C, Poulsen P, Simonsson S, Rönn T, Holmkvist J, Almgren P (2007). Genetic and epigenetic factors are associated with expression of respiratory chain component NDUFB6 in human skeletal muscle. J Clin Invest..

[126] Rönn T, Poulsen P, Hansson O, Holmkvist J, Almgren P, Nilsson P (2008). Age influences DNA methylation and gene expression of COX7A1 in human skeletal muscle. Diabetologia..

[127] Hernando-Herraez I, Evano B, Stubbs T, Commere P-H, Jan Bonder M, Clark S (2019). Ageing affects DNA methylation drift and transcriptional cell-to-cell variability in mouse muscle stem cells. Nat Commun..

[128] Conboy IM, Conboy MJ, Wagers AJ, Girma ER, Weissman IL, Rando TA (2005). Rejuvenation of aged progenitor cells by exposure to a young systemic environment. Nature..

[129] Davis RL, Weintraub H, Lassar AB (1987). Expression of a single transfected cDNA converts fibroblasts to myoblasts. Cell..

[130] Taylor SM, Jones PA (1979). Multiple new phenotypes induced in 10 T1/2 and 3 T3 cells treated with 5-azacytidine. Cell..

[131] Takahashi K, Yamanaka S (2006). Induction of pluripotent stem cells from mouse embryonic and adult fibroblast cultures by defined factors. Cell..

[132] Takahashi K, Tanabe K, Ohnuki M, Narita M, Ichisaka T, Tomoda K (2007). Induction of pluripotent stem cells from adult human fibroblasts by defined factors. Cell..

[133] Yu J, Vodyanik MA, Smuga-Otto K, Antosiewicz-Bourget J, Frane JL, Tian S (2007). Induced pluripotent stem cell lines derived from human somatic cells. Science..

[134] Hirano K, Nagata S, Yamaguchi S, Nakagawa M, Okita K, Kotera H (2012). Human and mouse induced pluripotent stem cells are differentially reprogrammed in response to kinase inhibitors. Stem Cells Dev..

[135] Ying Q-L, Wray J, Nichols J, Batlle-Morera L, Doble B, Woodgett J (2008). The ground state of embryonic stem cell self-renewal. Nature..

[136] Stearns-Reider KM, D’Amore A, Beezhold K, Rothrauff B, Cavalli L, Wagner WR (2017). Aging of the skeletal muscle extracellular matrix drives a stem cell fibrogenic conversion. Aging Cell..

[137] Biressi S, Miyabara EH, Gopinath SD, Carlig PM, Rando TA (2014). A Wnt-TGF 2 axis induces a fibrogenic program in muscle stem cells from dystrophic mice. Sci Transl Med.

[138] Pessina P, Kharraz Y, Jardí M, Fukada S, Serrano AL, Perdiguero E (2015). Fibrogenic cell plasticity blunts tissue regeneration and aggravates muscular dystrophy. Stem Cell Rep..

[139] Brack AS, Conboy MJ, Roy S, Lee M, Kuo CJ, Keller C (2007). Increased Wnt signaling during aging alters muscle stem cell fate and increases fibrosis. Science.

